# Advanced biomaterials for rare Krabbe disease: galactocerebrosidase scaffolds in demyelinating lesions

**DOI:** 10.1097/MS9.0000000000004240

**Published:** 2025-10-30

**Authors:** Ayesha Ashraf, Hiba Ashraf, Mahnoor Fatima, Muhammad Talha, Omar Abdullah Gill

**Affiliations:** a Department of Medicine, King Edward Medical University, Lahore, Pakistan; b Department of Medicine, Dow University of Health Sciences, Karachi, Pakistan; c Department of Medicine, Shaheed Ziaur Rahman Medical College, Rajshahi Medical University, Bogura, Bangladesh

*To the Editor*,

Krabbe disease, also known as globoid cell leukodystrophy, is a hereditary condition in which the body is unable to break down specific fats present in myelin efficiently due to the deficiency of galactosylceramidase (GALC) enzyme that degrades galactosylceramides in myelin membranes. With insufficient GALC, psychosine accumulates in oligodendrocytes and Schwann cells, resulting in central and peripheral nervous system demyelination. Its forms are classified as either infantile, juvenile, or adult-onset. The current treatment option available is hematopoietic stem cell transplantation, but it does not stop the progression of the disease. Monotherapies have been ineffective. However, recent studies show that combination treatment approaches could prove to be more effective^[1]^. This is in line with the TITAN Guidelines on the need for transparency in AI use in healthcare^[2]^.

Advanced biomaterials may be helpful in treating Krabbe disease by transporting functional GALC across the blood–brain barrier (BBB), where they can restore enzyme function and eliminate harmful psychosine before it damages myelin-producing cells. These nanoparticles provide a supportive environment for cells to recover and form new myelin, which cannot be achieved through traditional enzyme replacement therapies. They can also be used along with stem cell transplants or gene therapy to overcome the treatment challenges and improve longevity^[3]^.

Clinical studies have demonstrated that psychosine accumulates (~170 pmol/g tissue) within the lipid rafts of brains with Krabbe disease. This toxic sphingolipid then results in inhibition of protein kinase C signaling, altered cholesterol distribution, and disruption of protein markers^[4]^. Combination therapies that aim to increase GALC enzyme delivery to the CNS have contributed to extended lifespan, slower disease progression, and improved neurobehavioral outcomes^[5]^. Moreover, biological scaffolds have shown enhanced remyelination and functional recovery in other conditions involving demyelination, such as spinal cord injury and traumatic brain injury^[6]^.

One significant limitation is the complex microenvironment of Krabbe lesions, particularly ongoing neuroinflammation may reduce scaffold efficacy. In addition, the BBB can restrict systemic administration of these scaffolds, hence requiring invasive neurosurgical placement. This nature of procedures will be associated with high cost, potential patient reluctance, and the need for specialized neurosurgical expertise, limiting the availability.

Existing treatments for Krabbe disease, like hematopoietic stem cell transplantation, fail to stop the disease progression^[1]^. Therefore, the persistent psychosine accumulation and the limited ability to deliver enzymes to the brain highlight the need for better approaches^[4]^. These problems could be addressed using scaffolds loaded with GALC to enable targeted enzyme delivery, similar to successful uses in other demyelinating diseases^[6]^. However, comprehensive preclinical studies are essential to confirm their safety and effectiveness with a major focus on creating minimally invasive delivery techniques and refining scaffold designs to effectively cross the BBB^[3]^.

In conclusion, further studies are required to support the therapeutic efficacy of GALC-loaded scaffolds in Krabbe disease. If proven, this would be a significant advancement in disease treatment, potentially serving as an adjunct or replacement to HSCT in the future.

## Data Availability

None.
